# Protective effecs of baicalin magnesium on non-alcoholic steatohepatitis rats are based on inhibiting NLRP3/Caspase-1/IL-1*β* signaling pathway

**DOI:** 10.1186/s12906-023-03903-2

**Published:** 2023-03-06

**Authors:** Xiulu Guan, Shiyuan Shen, Jinxia Liu, Hongru Song, Jinhua Chang, Xiaoxia Mao, Jingyu Song, Lin Zhang, Cuizhe Liu

**Affiliations:** 1 Hebei Province Key Laboratory of Research and Development for Chinese Medicine, Institute of Traditional Chinese Medicine, Chengde Medical College, Anyuan Road, Shuangqiao District, Chengde, 067000 Hebei Province China; 2 Heibei North University, Zhangjiakou, 075000 China

**Keywords:** Baicalin magnesium, Non-alcoholic steatohepatitis, Inflammation, Lipid accumulation, Oxidative stress

## Abstract

Baicalin magnesium is a water-soluble compound isolated from the aqueous solution by *Scutellaria baicalensis* Georgi. Preliminary experiments have demonstrated that baicalin magnesium can exert protective effects against acute liver injury in rats induced by carbon tetrachloride or lipopolysaccharide combined with d-galactose by regulating lipid peroxidation and oxidative stress. The aim of this study was to investigate the protective effect of baicalin magnesium on non-alcoholic steatohepatitis (NASH) in rats and to elucidate the underlying mechanisms. NASH was induced through a high-fat diet (HFD) for 8 weeks, and Sprague-Dawley rats were intravenously injected with baicalin magnesium, baicalin, and magnesium sulfate for 2 weeks, respectively. Serum was obtained for biochemical analyses and the determination of oxidative stress indicators. Liver tissues were collected for use in liver index assessment, histopathological examination, inflammatory factor analysis, and protein and gene expression analysis. The results revealed that baicalin magnesium markedly improved HFD-induced lipid deposition, inflammatory response, oxidative stress, and histopathological impairments. And baicalin magnesium may exert a protective effect on NASH rats by inhibiting the NLR family pyrin domain involving the 3 (NLRP3)/caspase-1/interleukin (IL)-1*β* inflammatory pathway. Additionally, the effect of baicalin magnesium was remarkably superior to that of equimolar baicalin and magnesium sulfate in regard to ameliorating NASH symptoms. In conclusion, the findings suggested that baicalin magnesium may represent a potential drug for the treatment of NASH.

**Supplementary Information:**

The online version contains supplementary material available at 10.1186/s12906-023-03903-2.

## Introduction

Non-alcoholic steatohepatitis (NASH) is an inflammatory subtype of non-alcoholic fatty liver disease (NAFLD) that can progress to end-stage liver diseases such as liver fibrosis, cirrhosis, and liver cancer that is a crucial pathological stage of chronic liver disease [1]. The incidence and mortality of NASH are increasing world-wide; however, there are currently no effective drug treatment strategies for this disease. NASH is mainly prevented and treated clinically by controlling diet and exercise [2], and the vast majority of patients have difficulty in regard to effectively losing weight and maintaining a healthy weight in the long term. Accordingly, an in-depth study of NASH therapeutic agents is of great significance for the prevention and treatment of this disease.

**Fig. 1:**
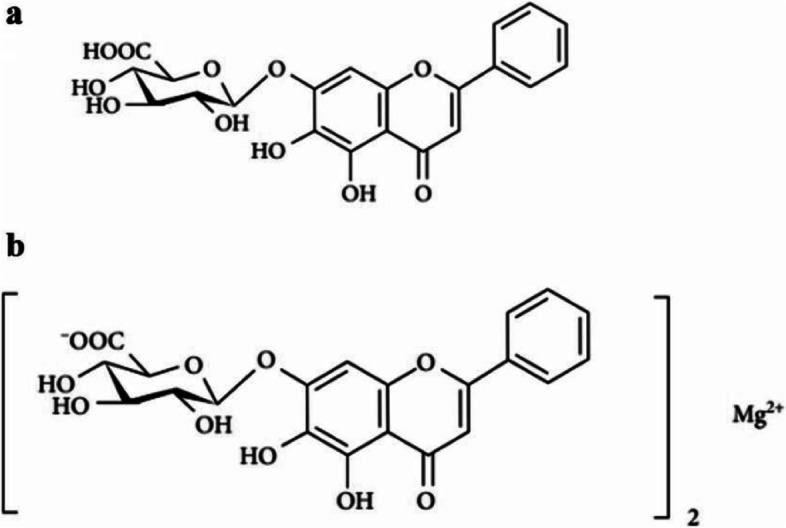
Chemical structures of **a** baicalin and **b** baicalin magnesium

*Scutellaria baicalensis* Georgi is a traditional Chinese medicine that is commonly used in the clinic [3]. Recently, *Scutellaria baicalensis* has been increasingly used in the mitigation and therapy of various clinical liver diseases. Clinically, *Scutellaria baicalensis* as the active ingredient of Shenqin compounded formulations and Shuganning injection can play a role in liver protection by increasing the secretion of hepatocyte growth factor, stimulating hepatocyte regeneration, scavenging oxygen free radicals and regulating immunity [4]. Baicalin is the primary effective component of *Scutellaria baicalensis*, which has been shown to have anti-inflammatory, antioxidant, anti-lipid deposition, and hepatoprotective effects in modern medical research [5–7]. Clinically, baicalin tablets or capsules can be used to treat chronic liver disease [8]. However, baicalin exhibits poor solubility and bioavailability, limiting its clinical application [9]. To solve this problem, we discovered that baicalin is currently produced by aqueous extraction and acid precipitation mainly according to the Chinese Pharmacopoeia, but the solubility of baicalin changes significantly before and after the addition of acid. So we have isolated a water-soluble baicalin from the aqueous decoction (without adding acid) of *Scutellaria baicalensis*, namely, baicalin magnesium, consisting of two molecules of baicalin and one molecule of magnesium ions, as illustrated in Fig. 1 [10]. The solubility of baicalin magnesium (200 mg/ml) was more than 3000 times that of baicalin (0.058 mg/ml). Our preliminary experiments suggested that baicalin magnesium had a protective effect against drug-induced acute liver injury (ALI) by modulating inflammatory responses and oxidative stress; moreover, the protective effect of baicalin magnesium on ALI was superior to that of baicalin [11–13]. Additionally, baicalin has favorable hypolipidemic effects [14]; however, whether baicalin magnesium can exert therapeutic effects on NASH rats by hypolipidemic effects and modulating of inflammatory responses and oxidative stress has not yet been previously investigated. Therefore, we investigated the therapeutic effects of baicalin magnesium on high-fat diet (HFD)-induced NASH.

The present study aimed to establish HFD-induced NASH rat models. Then the therapeutic effects of baicalin magnesium by tail vein injection on NASH in rats were examined according to pathological examination, biochemical indicators, inflammation, and oxidative stress. NLR family pyrin domain containing 3 (NLRP3) is a cytoplasmic multiprotein complex responsible for the activation of inflammatory reactions [15]. Activation of the NLRP3 inflammasome in hepatocytes has been demonstrated to be associated with the pathogenesis of liver diseases [16]. NLRP3 mediated the cleavage and maturation of interleukin (IL)-18 and IL-1*β*, and this in turn led to a complex network of cellular responses that triggers local and systemic inflammatory responses [17]. The protein and gene expression of NLRP3 and other related inflammatory cytokines in rats were assessed to evaluate the regulatory effects and mechanisms of baicalin magnesium in NASH rats.

## Materials and methods

### Reagents and chemicals

Baicalin (purity 76%, batch no. ZLSW2108090–1) was purchased from Xi’an Zelang Biotechnology Co., LTD (Xi’an, China). Magnesium sulfate (MgSO_4_, purity 98.0%, analytical purity, batch no. 20130812) was obtained from Sinopharm Chemical Reagent Co., LTD (Shanghai, China). Baicalin magnesium (purity 80.98%) was obtained from Professor Cuizhe Liu. High-fat feed (88% basic feed, 10% lard and 2% cholesterol) [18] was obtained from Beijing Hfk Bioscience Co., LTD (Beijing, China). ALT, aspartate aminotransferase (AST), total cholesterol (TC), triglyceride (TG), high-density lipoprotein cholesterol (HDL-C), and low-density lipoprotein cholesterol (LDL-C) kits were supplied by Ningbo Purebio Biotechnology Co., LTD (Ningbo, China). Myeloperoxidase (MPO), superoxide dismutase (SOD) and malonaldehyde (MDA) kits were obtained from Jiancheng Bioengineering Institute of Nanjing (Nanjing, China). IL-6 and IL-10 enzyme-linked immunosorbent assay (ELISA) kits were obtained from Cusabio Technology Co., LTD (Barksdale, USA). The IL-1*β* ELISA kit was purchased from Multi Sciences Biotechnology Co. LTD (Hangzhou, China). Antibodies against NLRP3, caspase-1, tumor necrosis factor (TNF-*α*), IL-18, and IL-1*β* were obtained from Proteintech Group, Inc. (Chicago, IL, USA). The rapid total RNA extraction kit was purchased from Hangzhou Haoke Biotechnology Co., LTD (Hangzhou, China).

### Animals

Forty-eight male Sprague-Dawley rats (2 ~ 3 weeks of age, 100 ~ 140 g) were purchased from Beijing Hfk Bioscience Co., LTD (License number: SCXK(Jing)2019–0008), and reared in Chengde Medical College Laboratory Animal Center (Certificate No.: SYXK [Ji] 2017–001) with conditions (22 ± 2 °C, 60 ± 5% humidity, and 12–12 h light-dark cycle). The animal experiments were reviewed and approved by the Ethics Committee of Chengde Medical College Laboratory Animal Center (Ethics No. LAC2021032). All methods were reported in compliance with ARRIVE guidelines for the reporting of animal experiments.

### Experimental protocol

**Fig. 2:**
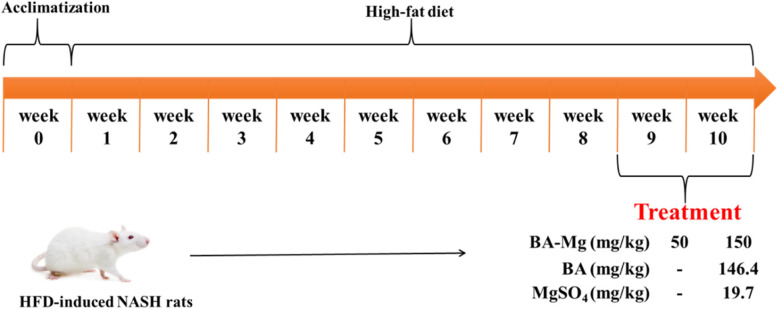
Timeline demonstrating the induction of NASH and treatment. (BA-Mg: baicalin magnesium; BA: baicalin)

As shown in Fig. 2, rats were randomly divided into six groups with eight rats in each group, including the control group, model group, baicalin magnesium groups (50 mg/kg and 150 mg/kg), baicalin group (146.4 mg/kg) and MgSO_4_ group (19.7 mg/kg). Baicalin magnesium and MgSO_4_ were dissolved in sterilized water for injection. Baicalin was added to sterilized water for injection and then adjusted to pH 7.4 with 10% NaOH. The concentrations of baicalin magnesium, baicalin and MgSO_4_ were 97 mg/ml, 188 mg/ml and 40 mg/ml, respectively. The baicalin group was administered a dose that was equimolar to the baicalin parent nucleus of baicalin magnesium (150 mg/kg), and the MgSO_4_ group was administered equimolarly to the magnesium ion of baicalin magnesium (150 mg/kg). The tail vein of the control and model groups were injected with the same amount of saline as the administered group. After 1 week of normal feeding adaptation, rats (with the exception of the control group) were fed with HFD for 8 weeks to develop NASH, followed by 2 weeks of continuous HFD feeding. The rats were anesthetized by intraperitoneal injection of 1% thiopental sodium (50 mg/kg) [19], and the livers were removed to calculate the liver index and the ratio of liver mass to body mass. Blood was also collected for subsequent experiments.

### Serum parameters analysis

The serum was obtained after centrifugation at 3000 r/min for 15 min. The levels of AST, ALT, TC, TG, HDL-C and LDL-C in the serum were determined using an automatic biochemical analyzer.

### Inflammatory cytokines determination

The liver tissues were cut up, ground in liquid nitrogen, made into liver tissue homogenate and then centrifuged, and the supernatant was taken to detect the levels of IL-6, IL-10 and IL-1*β* according to the instructions of ELISA.

### Measurement of MPO and SOD activity and of MDA content

The activities of MPO and SOD and also the content of MDA in serum were measured according to the instructions of the kits.

### Hemotoxylin and eosin staining

Liver tissues were fixed in formaldehyde solution for 48 h, embedded in paraffin, sliced, and stained with hematoxylin and eosin (HE). The degree of hepatic steatosis, inflammatory activity and balloon changes were observed under a light microscope. The NAFLD activity score (NAS) was calculated according to the consensus of a discordant pathological diagnosis of non-alcoholic fatty liver disease form a prospective multicenter study [20]. Specific scoring criteria were shown in Table 1.

**Table 1:** Non-alcoholic fatty liver disease activity scores

Points	Hepatocellular steatosis	Intralobular inflammation (Necrotic foci at 20x)	Ballooning of hepatocytes
0	< 5%	None	None
1	5–33%	<2pc	Rare
2	34–66%	2-4pc	Mostly
3	> 66%	>4pc	

### Oil red O staining

Liver sections were quickly fixed with 4% paraformaldehyde and then closed with Oil Red O solution for 10 min at room temperature. This was followed by hematoxylin staining. Finally, the tissue sections were sealed with glycerol gelatin and analyzed using light microscopy.

### Western blot analysis

Hepatic tissues were lysed in RIPA lysis buffer, and protein concentrations were measured using a BCA protein assay. Proteins were separated by 10% sodium dodecyl sulfate polyacrylamide gel electrophoresis (SDS-PAGE) and transferred to nitrocellulose membranes. The blots were subsequently incubated with rabbit anti-IL-1*β* (1:2000), rabbit anti-IL-18 (1:500), rabbit anti-NLRP3 (1:1000), rabbit anti-TNF-*α* (1:1000), rabbit anti-caspase-1 (1:1000), and primary antibodies at 4 °C overnight, and this was followed with treatment with horseradish peroxidase (HRP)-conjugated secondary antibodies (1:5000) for 1 h. The blots were visualized with an electrochemiluminescent system (Model Number: ChemiScope 6100, from Shanghai Qinxiang Scientific Instruments Co.) and quantified using the semi-quantification software Image J.

### Quantitative real-time PCR

Total RNA from the liver tissues was extracted using Trizol reagent and transcribed into cDNA. Briefly, quantitative real-time PCR was performed on an ABI Prism 7300 real-time PCR system. The target gene expression was quantified using the relative quantification comparative CT method. *β*-actin was used to normalize mRNA levels. The primer sequences used for real-time quantitative PCR were listed in Table [Table Tab2].

**Table 2 Tab2:** Primer sequences used for real-time PCR

Genes	Fowards (5′ > 3′)	Reverse (5′ > 3′)
TNF-*α*	ACTGGCGTGTTCATCCGTTCT	GCCACTACTTCAGCGTCTCG
IL-1*β*	AGCATCCAGCTTCAAATCTCAC	ACTAGCAGGTCGTCATCATCCC
Caspase-1	AACTGAACAAAGAAGGTGGCG	GCAAGACGTGTACGAGTGGGT
NLRP3	ACAGCCTTGAAGAGGAGTGGA	CTGGGTGTAGCGTCTGTTGAG
IL-18	CCCACAACGATGAGTACACCA	AGGATTACGGAAAGCATGGAG
*β*-actin	GTGTTGTCCCTGTATGCCTCTG	CTCTTTAATGTCACGCACGATTT

### Statistical analysis

Data analysis was performed using SPSS19.0, and all data were represented by the mean ± standard error of the mean (SEM). Data were analyzed by one-way analysis of variance (ANOVA) followed by Dunnett’s test, and differences were considered statistically significant at *P* < 0.05.

## Results

### Effect of baicalin magnesium on the pathological condition of NASH rats-represented by HE staining results

**Fig. 3:**
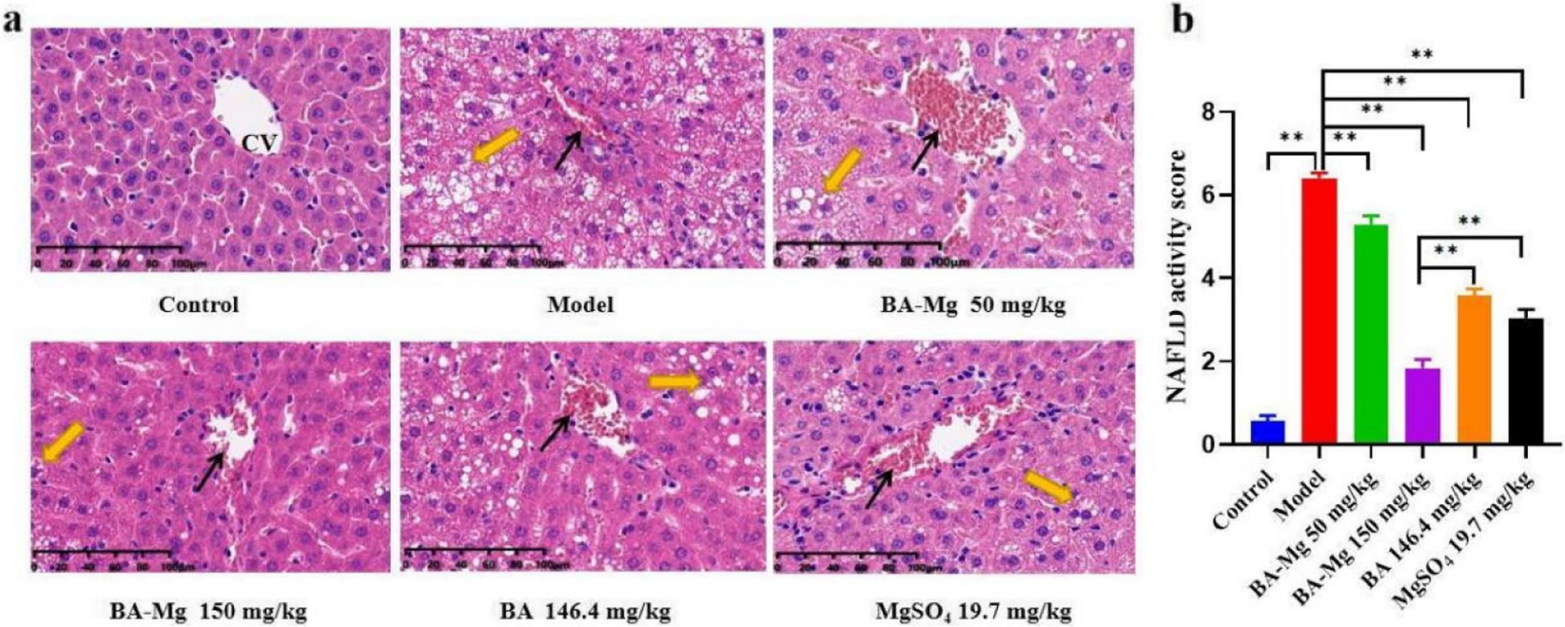
**a** HE staining and **b** NAS scores (*n* = 8). The black arrows represent inflammatory cells within the hepatic lobule, and the yellow arrows represent vacuolar-like changes within the hepatocytes, CV stands for central venous. (BA-Mg: baicalin magnesium; BA: baicalin. **P < 0.01)

Morphological changes in liver tissue were observed by HE staining, and the pathological status was scored as presented in Fig. 3. Compared to the control group, rats in the model group exhibited unclear nucleus boundaries, marked deformation of hepatocyte structures, liver inflammatory infiltration and necrosis, an obvious increase in balloon-like spots, and a significant increase in NAS (*P* < 0.01). Compared to the model group, liver inflammation was reduced in each administration group, the degrees of vacuolar changes and patchy necrosis were reduced, and NAS scores were lower (*P* < 0.05). The treatment effect of baicalin magnesium group (150 mg/kg) was significantly better compared to that of the baicalin and magnesium sulfate treatment groups (*P* < 0.01).

### Effect of baicalin magnesium on liver index and pathological conditions of NASH rats-represented by oil red O staining results

**Fig. 4:**
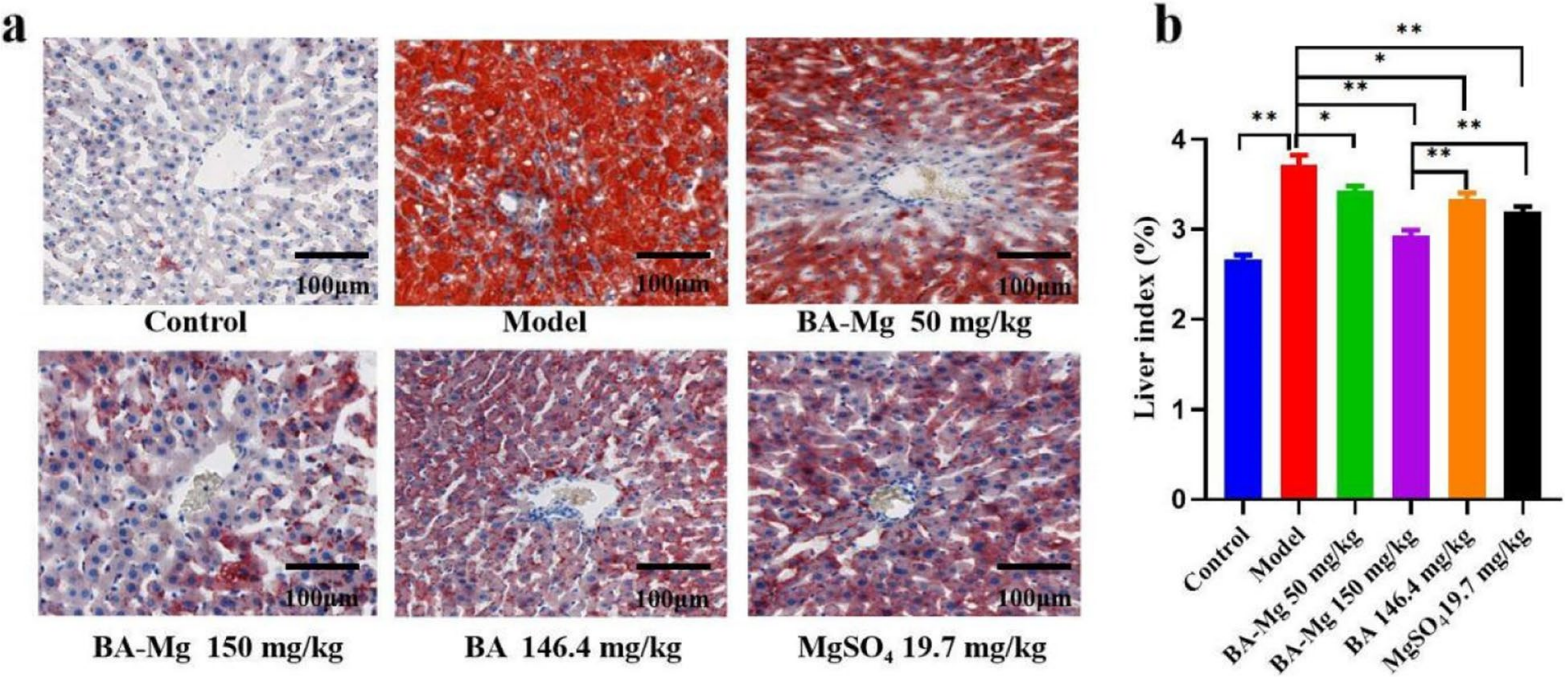
**a** Oil Red O staining and **b** liver index (*n* = 8). (BA-Mg: baicalin magnesium; BA: baicalin. *P < 0.05, **P < 0.01)

The liver index was calculated by measuring the body and liver weights as presented in Fig. 4b. The liver index in the model group was significantly higher than that in the control group (*P* < 0.01). The liver index in the administration group was significantly lower than that in the model group (*P* < 0.01). The therapeutic effect of baicalin magnesium was superior to that of equimolar baicalin and magnesium sulfate (*P* < 0.01), respectively.

As presented in Fig. 4a, the Oil Red O staining results revealed that lipid droplets in the model group were significantly increased but were decreased in the treatment groups, with the most overt decrease occurring in the baicalin magnesium group (150 mg/kg). These results suggested that baicalin magnesium can reduce lipid deposition in the liver of NASH rats.

### Improvement of biochemical indexes in the serum of NASH rats in response to baicalin magnesium

**Fig. 5:**
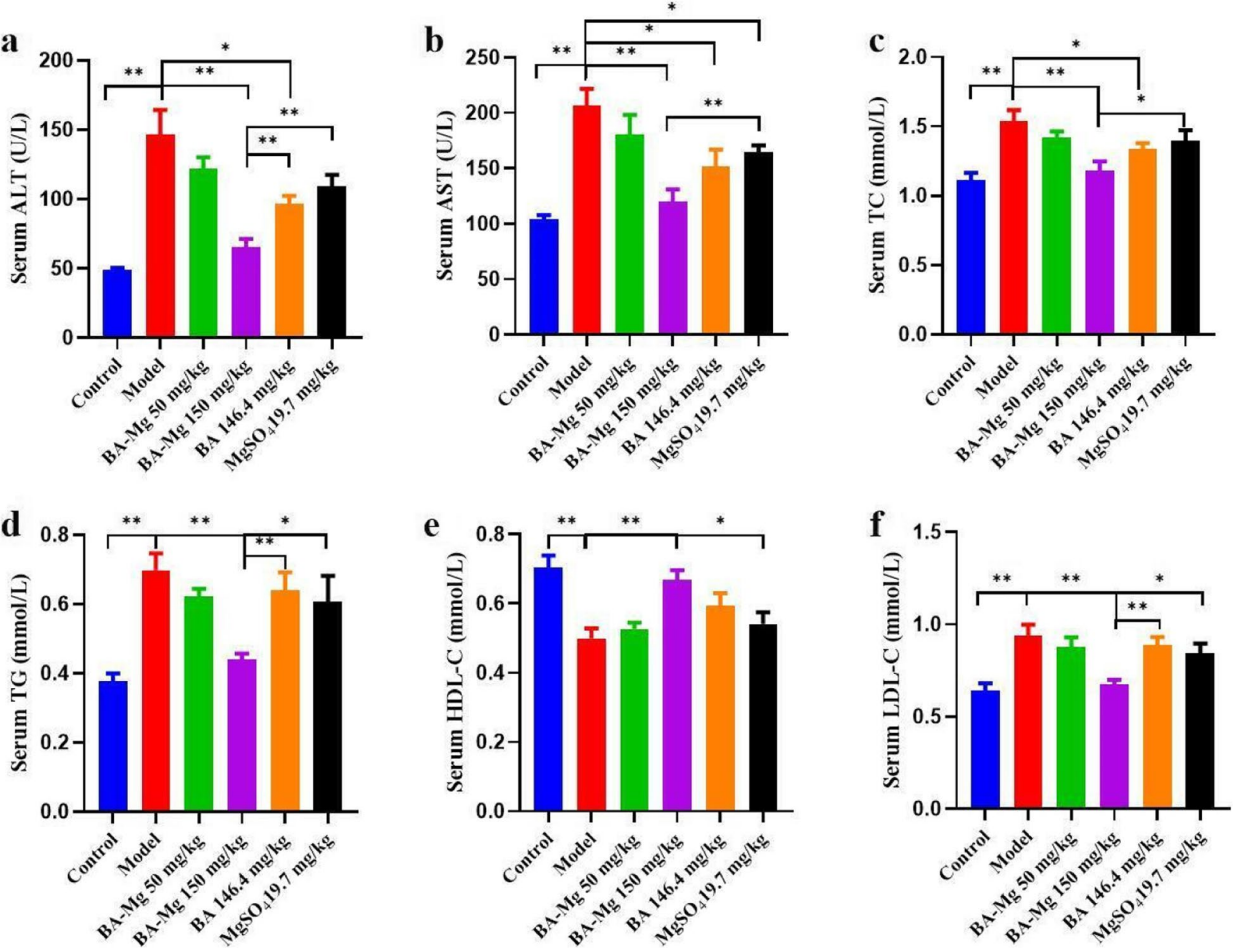
Effects of baicalin magnesium on biochemical indexes in the serum of NASH rats (*n* = 8). (BA-Mg: baicalin magnesium; BA: baicalin. *P < 0.05, **P < 0.01)

By measuring biochemical indices in the serum, we observed that baicalin magnesium can alleviate liver injury and lipid deposition in NASH rats. Compared to the control group, ALT, AST, LDL-C, TC, and TG levels in the model group were increased, while HDL-C levels were distinctly decreased (*P* < 0.01). As illustrated in Fig. 5, compared to the model group, ALT, AST, LDL-C, TC, and TG were decreased in all treatment groups, HDL-C was increased (*P* < 0.05), and the baicalin magnesium group (150 mg/kg) exhibited the most significant changes (*P* < 0.01).

### Effects of baicalin magnesium on SOD, MDA, and MPO in NASH rats

**Fig. 6:**
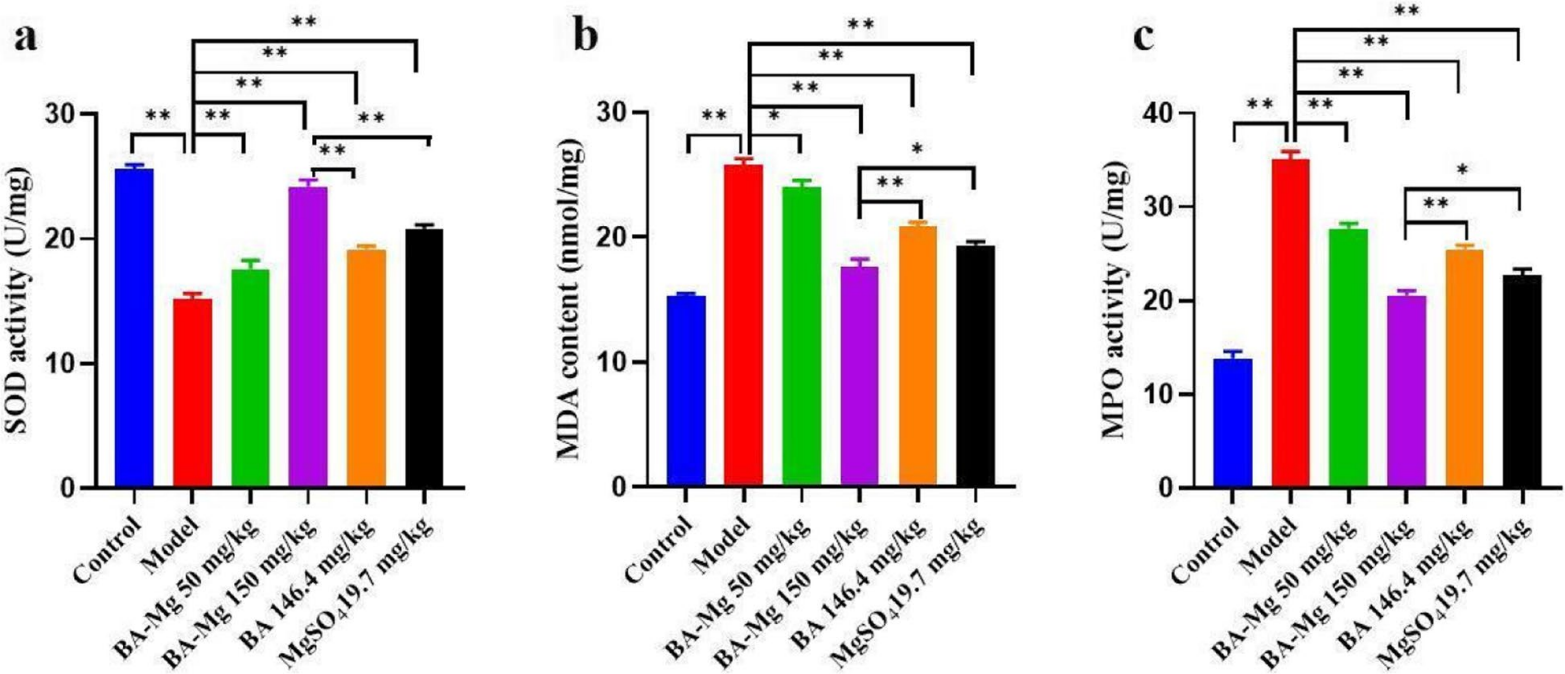
Effects of baicalin magnesium on **a** SOD activity, **b** MDA content and **c** MPO activity in the serum (*n* = 8). (BA-Mg: baicalin magnesium; BA: baicalin. *P < 0.05, **P < 0.01)

As illustrated in Fig. 6, SOD and MDA in the serum were used to explore the effects of baicalin magnesium on oxidative stress levels in NASH rats. Compared to the control group, SOD activity was significantly decreased, and MDA content was increased (*P* < 0.01). Compared to the model group, SOD activity was increased, and MDA content was significantly decreased (*P* < 0.01).

As presented in Fig. 6c, compared to the control group, MPO activity was increased (*P* < 0.01). Compared to the model group, MPO activity was decreased in all treatment groups, and the improvement effect in the baicalin magnesium group (150 mg/kg) was the most significant (*P* < 0.01).

### Baicalin magnesium can reduce the contents of IL-6, IL-1*β*, and increase the content of IL-10 in NASH rats

**Fig. 7:**
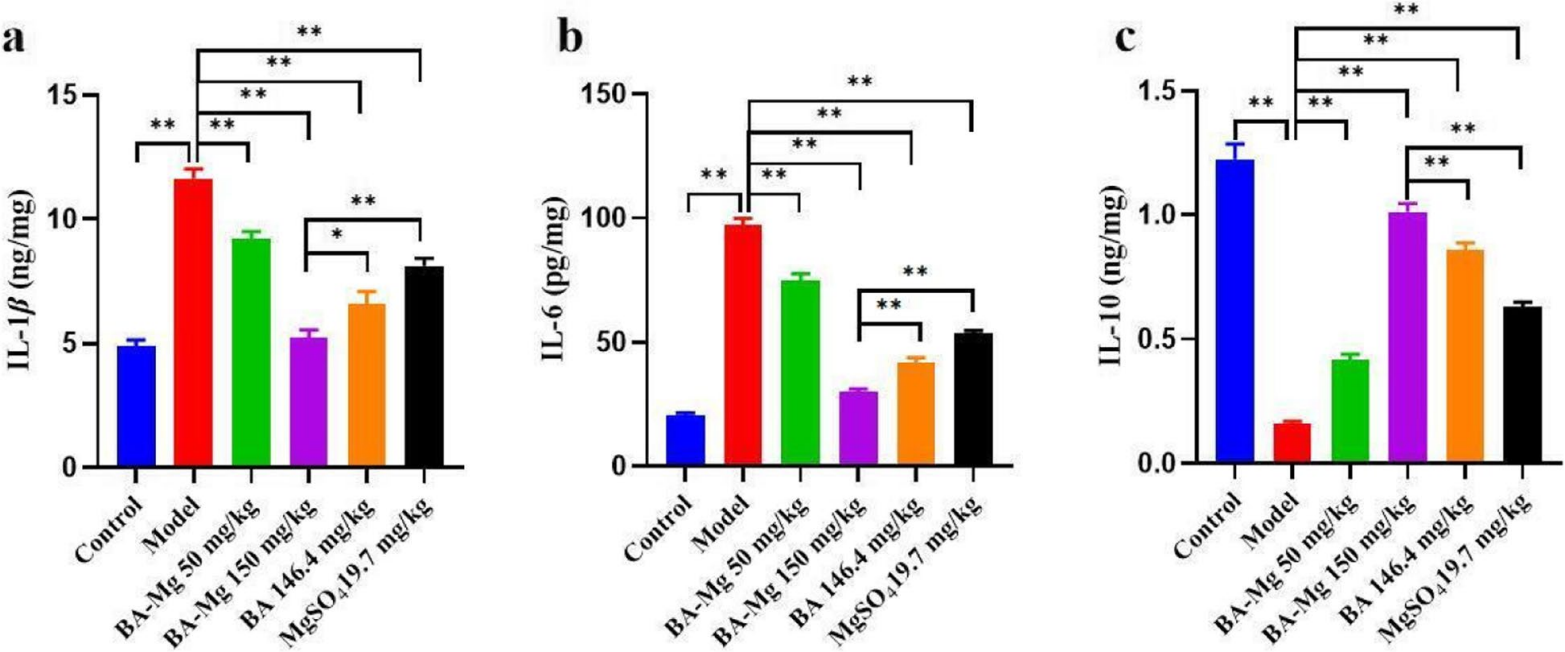
Effects of baicalin magnesium on inflammatory factors in liver tissue (*n* = 8). (BA-Mg: baicalin magnesium; BA: baicalin. *P < 0.05, **P < 0.01)

As presented in Fig. 7, compared to the model group, the levels of IL-6 and IL-1*β* decreased in all drug administration groups, while IL-10 was increased (*P* < 0.01). After treatment with baicalin magnesium, the levels of anti-inflammatory factors increased while those of pro-inflammatory factors decreased in the serum, thus indicating that baicalin magnesium can reduce the levels of inflammatory cytokines in NASH rats.

### Baicalin magnesium inhibits the inflammatory factor protein expression related to the NLRP3/caspase-1/IL-1*β* pathway

**Fig. 8:**
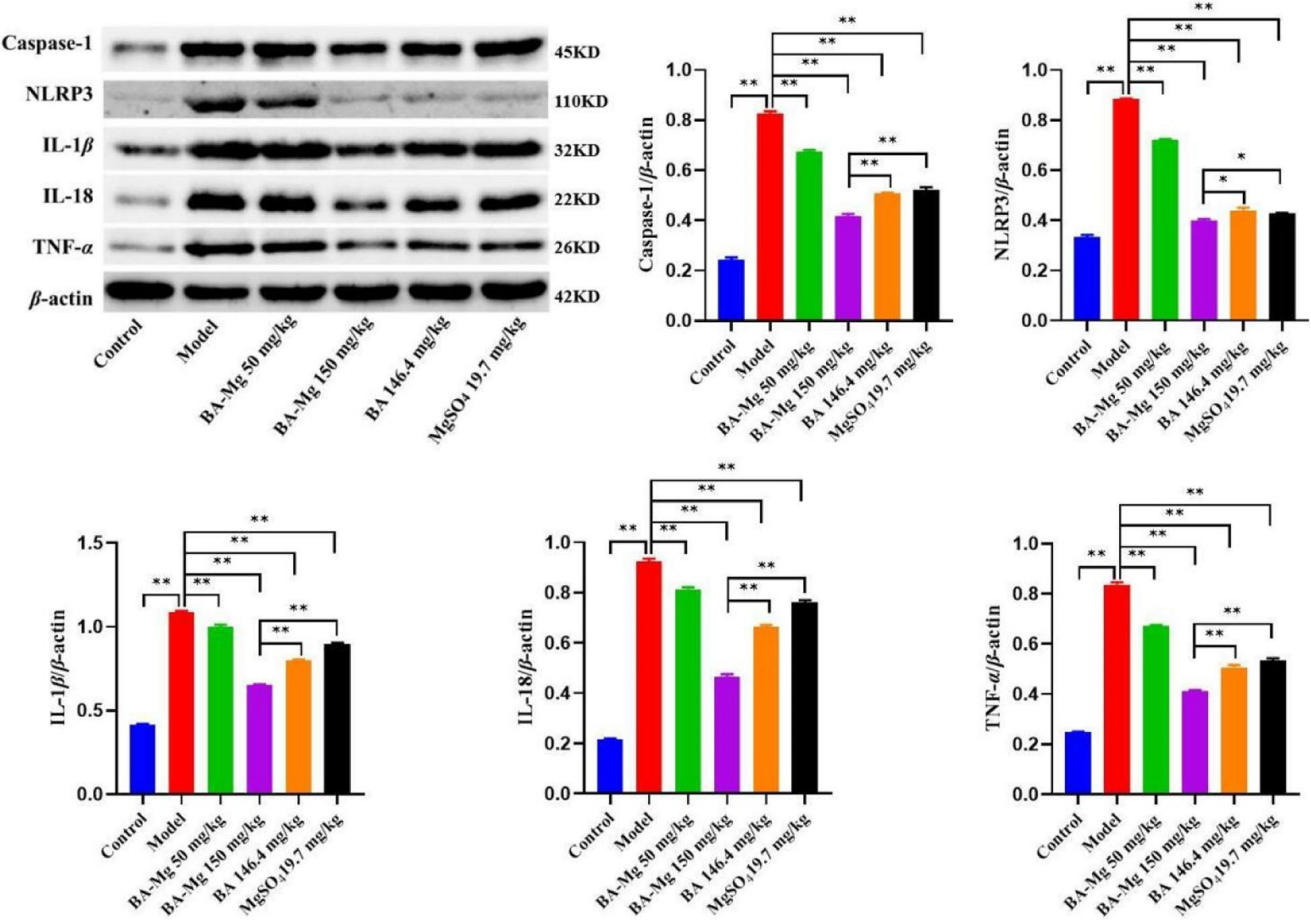
Effects of baicalin magnesium on the protein expression of caspase-1, NLRP3, IL-1*β*, IL-18 and TNF-*α* in the liver (*n* = 8). The bar graph represents the ratio of the gray value of the destination strip to the gray value of the inner reference strip. (BA-Mg: baicalin magnesium; BA: baicalin. *P < 0.05, **P < 0.01). Note: This image has been cropped to improve clarity and brevity, and all samples were from the same experiment

As presented in Fig. 8, compared to the control group, the expression of NLRP3, TNF-*α*, caspase-1, IL-18 and IL-1*β* in the model group was increased (*P* < 0.01). Compared to the model group, the protein expression of inflammatory factors in each administration group were decreased to varying degrees, while the effects of baicalin magnesium (150 mg/kg) were the most significant (*P* < 0.01).

### Baicalin magnesium inhibits the gene expression of inflammatory factors related to the NLRP3/caspase-1/IL-1*β* pathway

**Fig. 9:**
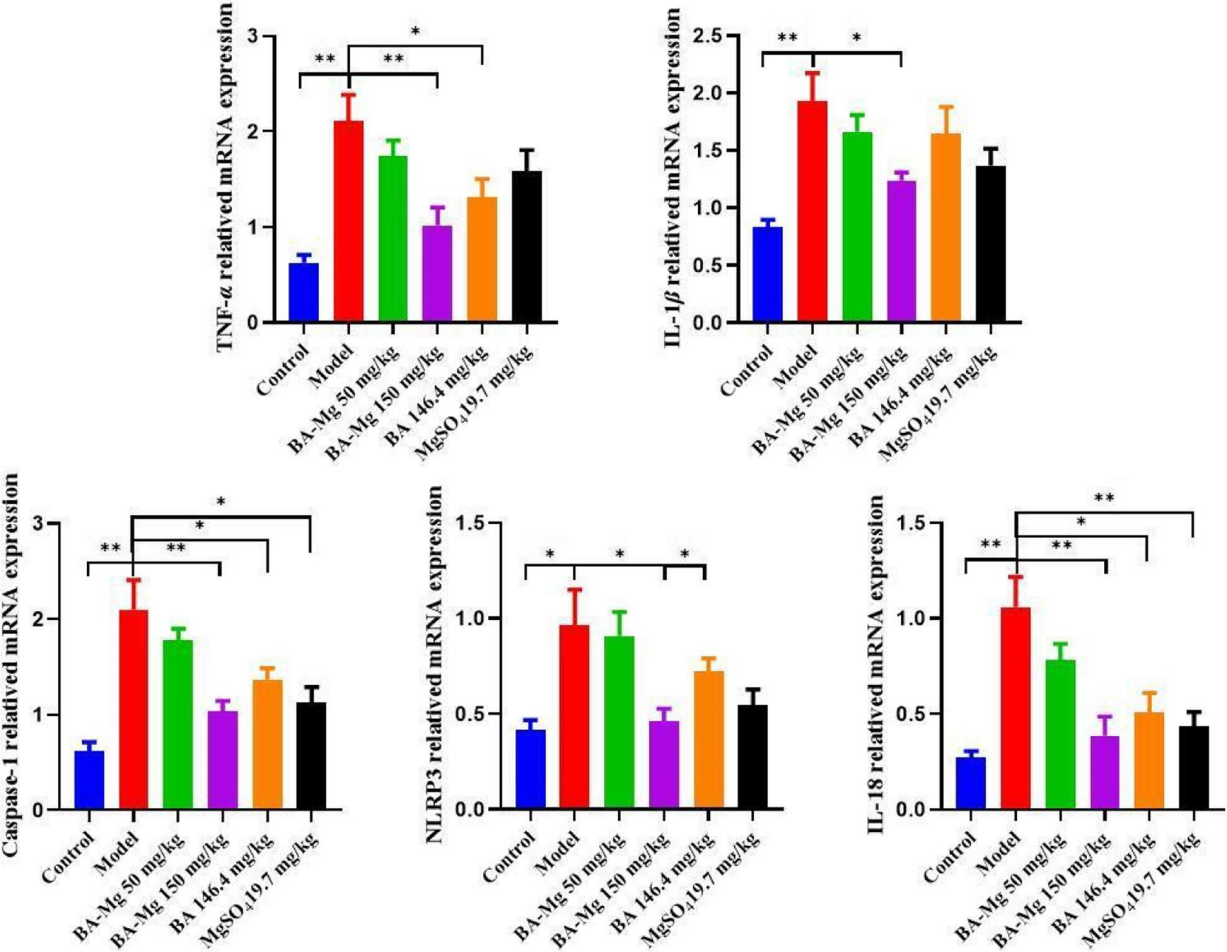
Effects of baicalin magnesium on the gene expression of TNF-*α*, IL-1*β*, caspase-1, NLRP3 and IL-18 in the liver (*n* = 8). (BA-Mg: baicalin magnesium; BA: baicalin. *P < 0.05, **P < 0.01)

As presented in Fig. 9, compared to the control group, the gene expression of NLRP3, TNF-*α*, caspase-1, IL-18, and IL-1*β* in the model group was increased (*P* < 0.01). Compared to the model group, the gene expression of inflammatory factors in the treatment groups was significantly decreased (*P* < 0.01), while the decrease of baicalin magnesium (150 mg/kg) were more marked.

## Discussions

The pathogenesis of NASH is complex and involves pathological conditions such as lipid metabolism disorders, insulin resistance, increased hepatic lipid peroxidation, and abnormal inflammatory cytokines [21, 22]. The dietary model is the most extensively used NASH model, and HFD-induced NASH can cause weight gain, obesity, and insulin resistance [23]. Accordingly, an HFD-induced rat model was used to conduct an in-depth study of NASH. The Chinese herb *Scutellaria baicalensis*, which can antipyretic-detoxicate [24], and its main active ingredient baicalin has been shown to play a role in regulating lipid metabolism, inflammation and oxidative stress responses. Zhang et al. found that baicalin significantly inhibited hepatic TG and TC content in MCD-induced NASH mice, reduced hepatic lipid deposition, and improved hepatic steatosis by regulating key factors of lipids such as CPT 1a [25]. Dai et al. found that baicalin has unique antisteatosis activity and that long-term treatment with baicalin improves diet-induced obesity and hepatic steatosis [14]. Liu et al. reported that baicalin reduced methionine-choline deficiency (MCD)-induced plasma transaminase, liver cell apoptosis, liver lipid deposition, and inflammatory cell infiltration by inhibiting toll-like receptor 4 (TLR4) signaling and inflammatory mediator production in NAFLD mice [26]. Ai et al. determined that baicalin could reduce the formation of fatty acid-binding proteins under oxidative stress, enhance the expression of the antioxidant enzyme SOD, and protect the liver cells from oxidative stress [27]. Baicalin improved body weight and hepatic lobule steatosis, thus suggesting that it could alleviate fatty degeneration and inflammation in NASH rats [28]. Zhang et al. observed that baicalin reduces serum TNF-*α*, IL-1*β* levels, monocyte chemoattractant protein-1 (MCP-1) production, macrophage influx, and nuclear factor-*κ*B (NF-*κ*B) activation, thus inhibiting dietary NASH symptoms induced by MCD [25]. In addition to, it has been found that reduced levels of magnesium ions in serum and liver lead to reduced mitochondrial function in the body, metabolic disorders, oxidative stress and inflammatory responses thus leading to liver-related diseases [29]. It was found that magnesium ion deficiency can lead to increased blood lipid levels as well as exacerbate lipid deposition in the liver [30]. Majid et al. revealed that magnesium supplementation had an ameliorating effect on the levels of ALT and AST, etc. in NASH patients, which had a protective effect on the liver [31]. Notably, the serum biochemical indexes and oil red O staining results showed that baicalin magnesium reduced lipid deposition better than baicalin and MgSO_4_, which may be due to the synergistic effect of baicalin and magnesium ions in the structure of baicalin magnesium, but the exact mechanism needs to be further investigated.

Inflammation plays an essential role in the activation of NASH-related cellular molecules and their initiation followed by damage to tissue cells [32–34]. It has been demonstrated that NLRP3 inflammatory vesicles were extensively involved in host immunity, inflammatory response and the development of chronic degenerative diseases. Not only would NLRP3 activate and initiate the inflammatory response, but also directly damage tissue cells, causing metabolic disorders and lipid deposition, etc. [35], which in turn further activates NLRP3. The activated NLRP3 recruits to pro-caspase-1, leading to the activation of caspase-1 [36], then the activated caspase-1 processes pro-IL-1*β* and pro-IL-18 for cleavage and processing, forming mature IL-1*β* and IL-18 and releasing them extracellularly [37, 38], exacerbating the inflammatory response [39–42], thus promoting the development and progression of NASH. TNF-*α* is capable of participating in systemic inflammatory responses and can transmit information to the nucleus through specific receptors on cell membranes, resulting in complex biological activities. This project reveals that lipid deposition in the liver of NASH rats activates the protein and gene expression of NLRP3 and its downstream inflammatory factors, intensifying the inflammatory response. Baicalin magnesium can improve the disorder of lipid metabolism in NASH rats, reduce hepatic lipid deposition, downregulate the protein and gene expression of TNF-*α,* NLRP3, caspase-1, IL-18 and IL-1*β*. Apart from that baicalin magnesium can effectively reduce the levels of pro-inflammatory factors IL-1*β* and IL-6, and increased anti-inflammatory factor IL-10. The above results indicated that baicalin magnesium can inhibit the inflammatory response and exert therapeutic effects on NASH rats by inhibiting the activation of NLRP3/caspase-1/IL-1*β* inflammatory pathway.

Liver lipid deposition induces an inflammatory response, and inflammation also further leads to liver damage, lipid deposition, and oxidative stress [43]. MPO can cause peroxidation at the site of liver inflammation, leading to hepatocyte damage [44]. ALT and AST are common indicators of liver injury. It was confirmed that baicalin magnesium could reduce MPO activity, ALT and AST levels, improving the degree of liver injury in NASH rats. Oxidative stress increases the release of lipid peroxides and inflammatory cytokines, and promotes the development of NASH. SOD and MDA are widely recognized indices used to evaluate lipid peroxidation [45], SOD is an important antioxidant enzyme in vivo that reflects the antioxidant capacity of the body and the inflammation level [39, 44]. MDA is a metabolite of lipid peroxidation, and its content reflects the degree of oxidative damage to the body to a certain extent [40]. It was shown that baicalin magnesium could improve the activity of SOD and the content of MDA, suggesting that it could remarkably improve the antioxidant capacity of NASH rats (*P* < 0.01). The pathological results also further confirmed that baicalin magnesium could effectively improve liver cell disorder, vacuole-like degeneration and damage, significantly reduce the number of lipid droplets and inflammatory cell infiltration, and have excellent anti-lipid deposition, anti-inflammatory and antioxidant effects in NASH rats.

## Conclusion

**Fig. 10:**
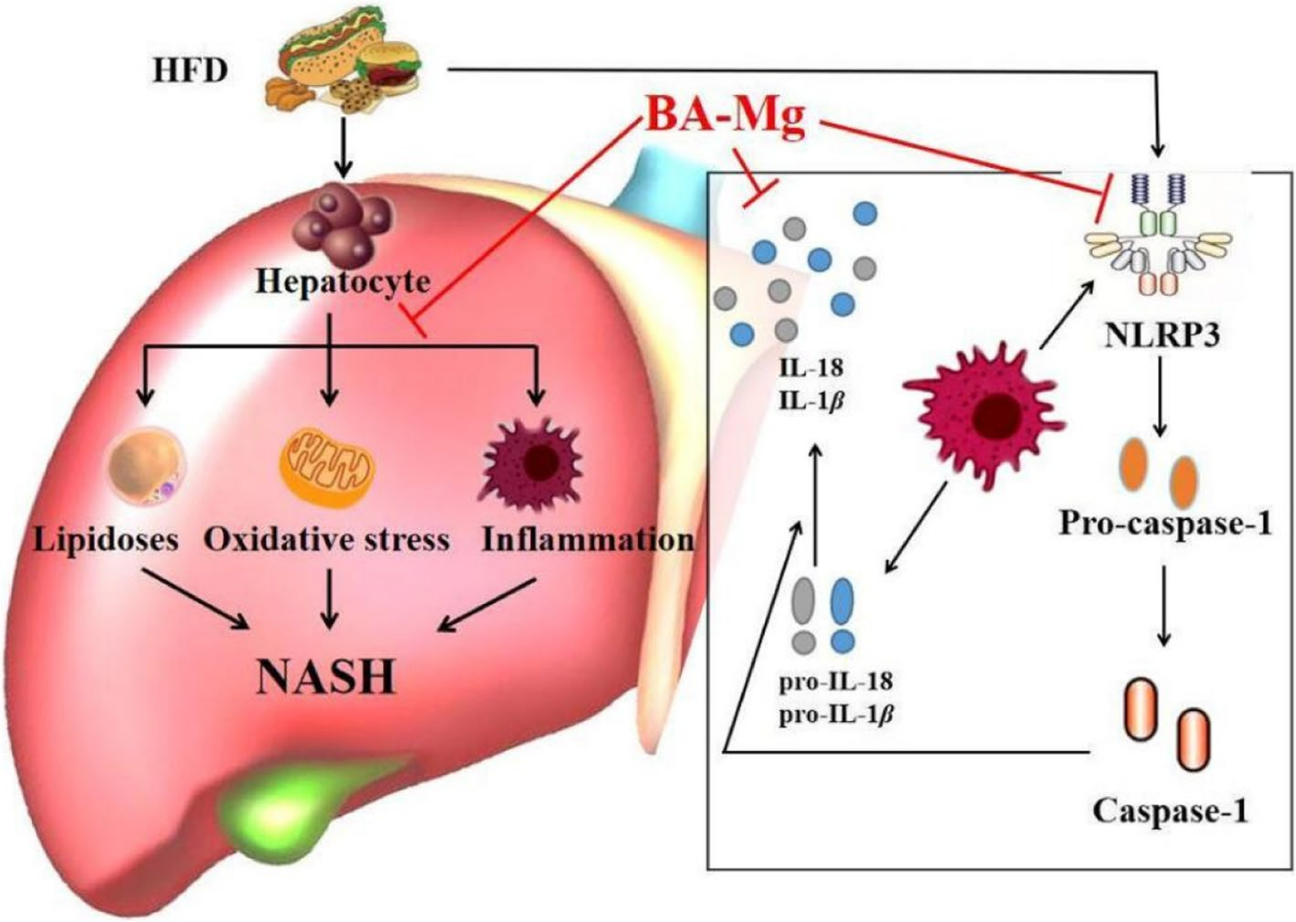
The mechanism of baicalin magnesium in the treatment of HFD-induced NASH rats

In conclusion, as shown in Fig. 10, baicalin magnesium has a therapeutic effect on HFD-induced NASH rats, which may act by reducing lipid deposition and oxidative stress, down-regulating the expression of NLRP3 inflammatory vesicles, and inhibiting the expression of caspase-1 and IL-18. Consequently, as a new drug baicalin magnesium possesses a promising future for the treatment of NASH.

## Supplementary Information

**Additional file 1.**

## Data Availability

The datasets used and analysed during the current study available from the corresponding author on reasonable request.
