# Supplementary material for: Protective effecs of baicalin magnesium on non-alcoholic steatohepatitis rats are based on inhibiting NLRP3/Caspase-1/IL-1β signaling pathway
Source: BMC Complement Med Ther. 2023 Mar 6;23:72. doi: 10.1186/s12906-023-03903-2 (PMC9987046; doi:10.1186/s12906-023-03903-2)

We have provided some additions for the above western blot experimental pictures. The supplemental file contained as full as possible length gels and blots with membrane edges visible. These images were the original, unprocessed versions.

The blots were visualized with an electrochemiluminescent system (Model Number: ChemiScope 6100, from Shanghai Qinxiang Scientific Instruments Co.) and quantified using the semi-quantification software Image J.

The following are additional figures.

***β*-actin**


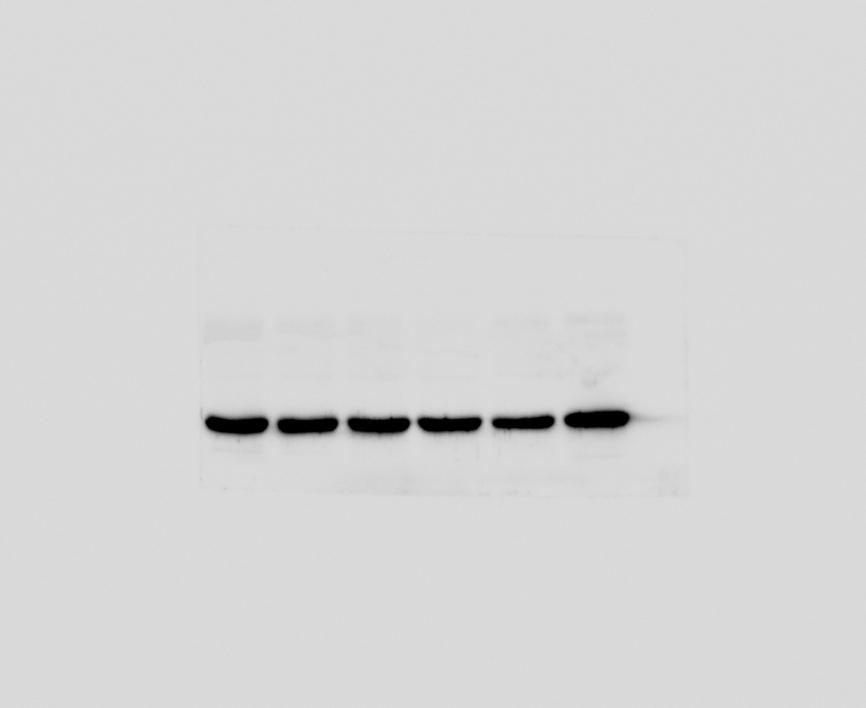


**Caspase-1**


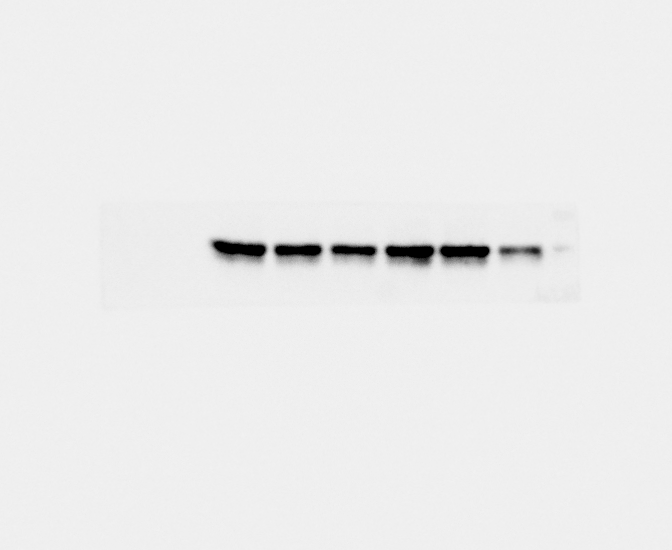


**NLRP3**


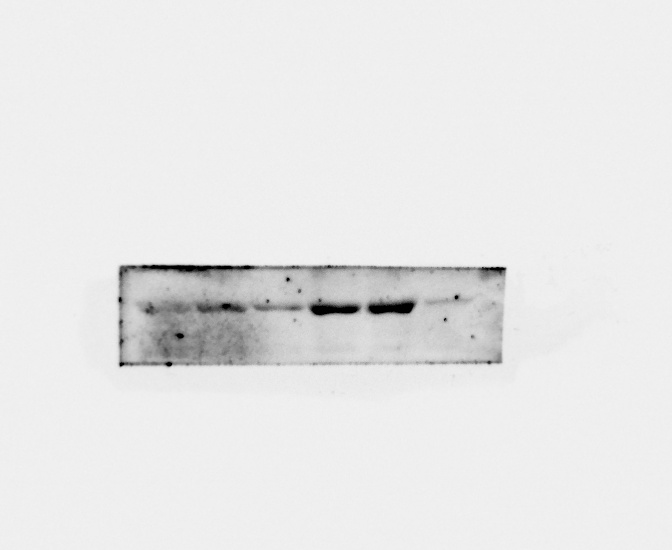


**Il-1*β***


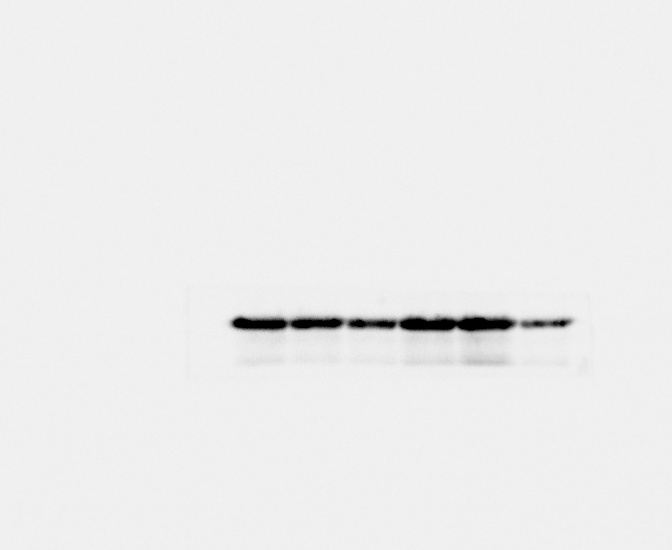


**IL-18**


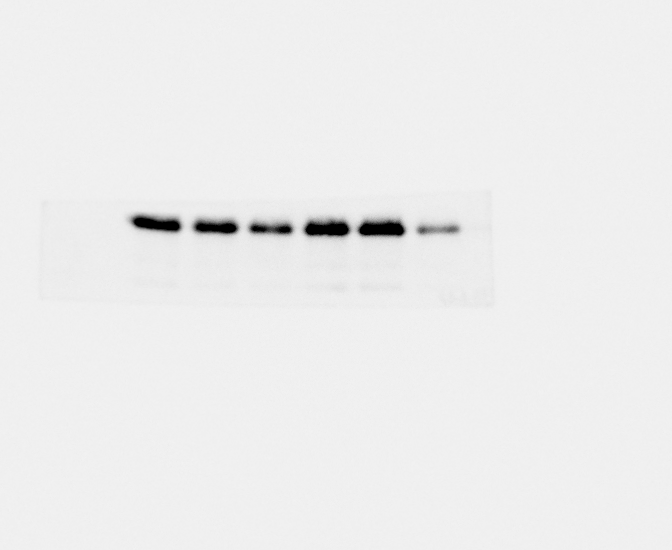


**TNF-*α***


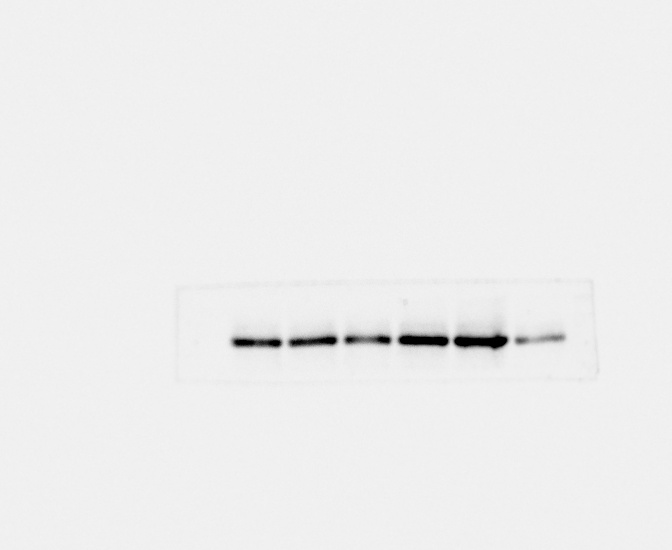

Supplement: Supplementary file 1 — Additional file 1. [file 12906_2023_3903_MOESM1_ESM.docx]
